# Chloroplast localization of *Cry1Ac* and *Cry2A* protein- an alternative way of insect control in cotton

**DOI:** 10.1186/s40659-015-0005-z

**Published:** 2015-03-13

**Authors:** Adnan Muzaffar, Sarfraz Kiani, Muhammad Azmat Ullah Khan, Abdul Qayyum Rao, Arfan Ali, Mudassar Fareed Awan, Adnan Iqbal, Idrees Ahmad Nasir, Ahmad Ali Shahid, Tayyab Husnain

**Affiliations:** National Center of Excellence in Molecular Biology, University of the Punjab, Lahore, 53700 Pakistan; Institute of Molecular Biology, Academia Sinica, Taipei, 115 Taiwan

**Keywords:** Chloroplast transient peptide, *Cry1Ac*, *Cry2A*, Bt, cTP, Cry genes, Endotoxins, *GUS*, Transgenic plants

## Abstract

**Background:**

Insects have developed resistance against Bt-transgenic plants. A multi-barrier defense system to weaken their resistance development is now necessary. One such approach is to use fusion protein genes to increase resistance in plants by introducing more Bt genes in combination. The locating the target protein at the point of insect attack will be more effective. It will not mean that the non-green parts of the plants are free of toxic proteins, but it will inflict more damage on the insects because they are at maximum activity in the green parts of plants.

**Results:**

Successful cloning was achieved by the amplification of *Cry2A*, *Cry1Ac*, and a transit peptide. The appropriate polymerase chain reaction amplification and digested products confirmed that *Cry1Ac* and *Cry2A* were successfully cloned in the correct orientation. The appearance of a blue color in sections of infiltrated leaves after 72 hours confirmed the successful expression of the construct in the plant expression system. The overall transformation efficiency was calculated to be 0.7%. The amplification of *Cry1Ac*-*Cry2A* and *Tp2* showed the successful integration of target genes into the genome of cotton plants. A maximum of 0.673 μg/g tissue of *Cry1Ac* and 0.568 μg/g tissue of *Cry2A* was observed in transgenic plants. We obtained 100% mortality in the target insect after 72 hours of feeding the 2^nd^ instar larvae with transgenic plants. The appearance of a yellow color in transgenic cross sections, while absent in the control, through phase contrast microscopy indicated chloroplast localization of the target protein.

**Conclusion:**

Locating the target protein at the point of insect attack increases insect mortality when compared with that of other transgenic plants. The results of this study will also be of great value from a biosafety point of view.

## Background

The discovery of the insecticidal effects of *Bacillus thuringiensis* in the early 20^th^ century has allowed for the development of new pest insect control methods. The Cry proteins solubilize in alkaline pH (9–12) following ingestion, and protoxins are then released. The protoxins are activated by specific enzymes in the midgut and bind to specific receptors in the microvilli of columnar cell apical membranes in lepidopteran insects [1]. The effect of Bt proteins is highly specific to certain insect species, and they are nontoxic to beneficial insects and animals [2]. Their relative safety for the environment, animals, humans, fishes, birds, and beneficial entomofauna is of great significance [3].

Transformation of these crystal protein (Bt) genes in plants, especially cotton, has been carried out for many years [4]. This limits the application of environmentally devastating pesticides. *Bacillus thuringiensis* (Bt) crystal proteins have attracted extensive attention as insecticidal molecules [5]. The reduction in pesticide application, up to 70%, has been documented in Bt cotton fields in India resulting in a saving of up to US$30 per ha in insecticide costs and an 80–87% increase in harvested cotton yield [6].

Cloning and transformation of various Bt genes have been done in higher plants but the resulting transgenic plants show lower insecticidal activity as insects develop Bt resistance in response to the level of gene expression [7]. Low toxin levels are of huge concern nowadays. To overcome this issue, several strategies have been employed by researchers, e.g., inserting the gene into the chloroplast genome [8,9], modifying the coding sequences of the bacterial gene to plant-preferred coding sequences [10], and expressing the genes in the chloroplast using chloroplast transient peptides [11].

The new trend in transformation for localized transgene expression is chloroplast transformation [11]. This technique is very useful in expressing genes in the green parts of the plants but its application has been limited to the *Solanaceae* family [12]. Most of the work on chloroplast transformation has concentrated on tobacco because it is easy to regenerate on tissue culture media following biolistic/agrobacterium transformation [13]. However, the recalcitrant nature of cotton plants makes them impossible to regenerate on tissue culture media [14], seriously hindering the application of chloroplast transformation technology in this plant. Though the chloroplast contains its own DNA, it only codes 10% of the required protein. The rest of the proteins are imported from the cytosol to the chloroplast through specific trans-peptide (TP) signals [15] having an N-terminal extension responsible for carrying the proteins to the organelle [16]. Based on this, cotton nuclear transformation might be achieved by tagging TP at the Bt gene N-terminal to transport precursor proteins into the chloroplast [17,18].

Several reports have confirmed that lepidopteran insects develop some resistance to Bt crops with a single Cry gene. Therefore, there is a need to develop new strategies comprising multiple lines of defense to cope with this developing resistance in insects [11]. The present study focused on two aspects; first, developing resistance in plants using two genes, i.e., *Cry1Ac* and *Cry2A*, and second, achieving the benefits of chloroplast targeted expression through nuclear transformation in cotton, where tissue culture on media is impossible. A higher production of target proteins can be achieved when the genes are expressed in plant chloroplasts [17,19,20] because when the transgene is stably integrated, plastid transformation accumulates large amounts of foreign proteins (up to 46% of total leaf protein) [21]. The higher expression is the result of thousands of copies of the chloroplast genome in each plant cell, which results in high copy numbers of the functional genes [22]. Other advantages that have been seen in chloroplast transgenic plants include a 169-fold increase in transgene expression compared with nuclear transformation and a lack of transgene silencing [23]. Another advantage of chloroplast targeted engineering includes transgene stacking, i.e. simultaneous expression of multiple transgenes, thus creating multivalent vacancies in a single transformation step [22].

The present study aimed to clone *Cry1Ac* and *Cry2A* genes and transit peptides with their fusion protein, which can localize its expression in the chloroplast. This study was designed for the production of modern transgenic cotton plants with minimal biosafety concerns. The transgene is expressed only in the green tissues because the fusion-protein gene attaches to Bt on C-terminal and cTP on N-terminal resulting in higher expression levels, which enhances lepidopteran insect resistance.

## Results

### Construction of the plant expression vector MUZ_01

**Figure 1:**
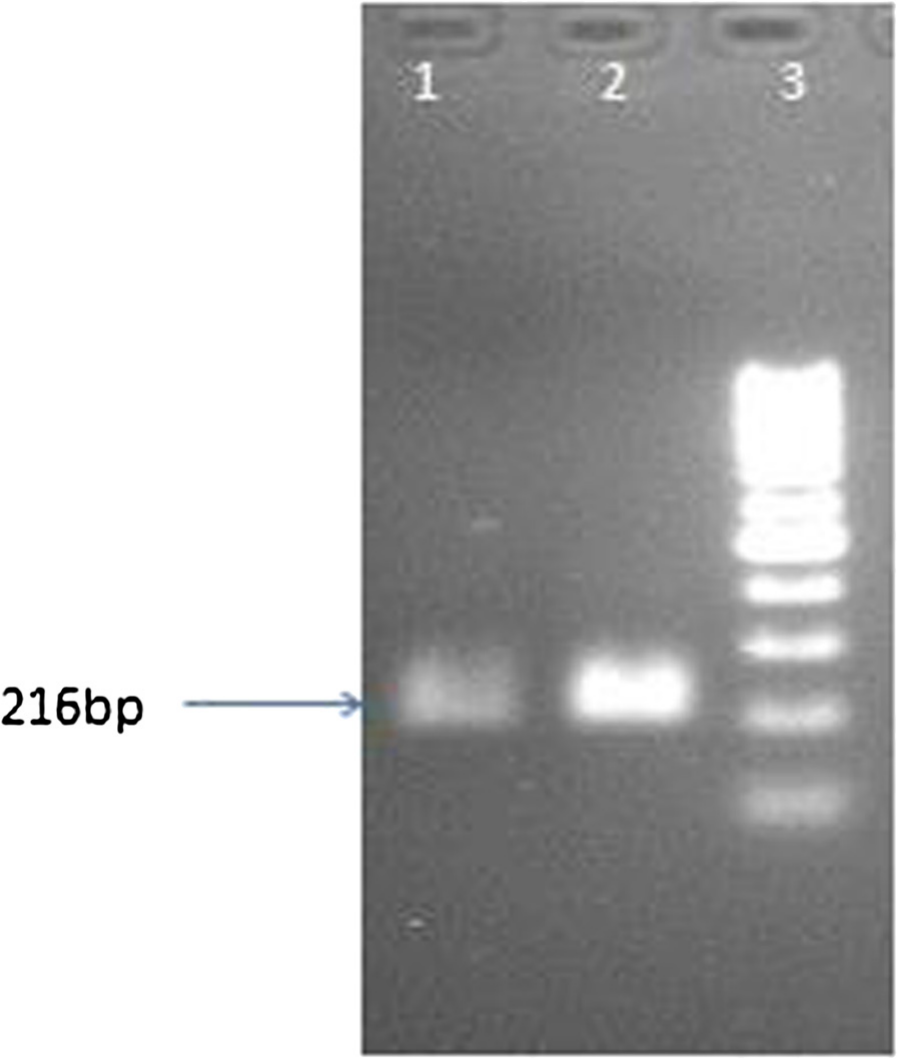
**Lane 1 and 2 show PCR product of 216 bp with full length primers of cTP while lane 3 is 1 kb ladder.**

**Figure 2:**
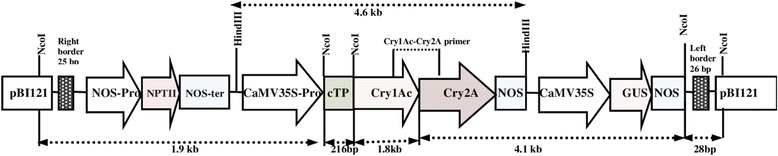
**Construct map (pBI-121-Tp-** ***Cry1Ac*** **-** ***Cry2A*** **-Nos) along with restriction sites.**

**Figure 3:**
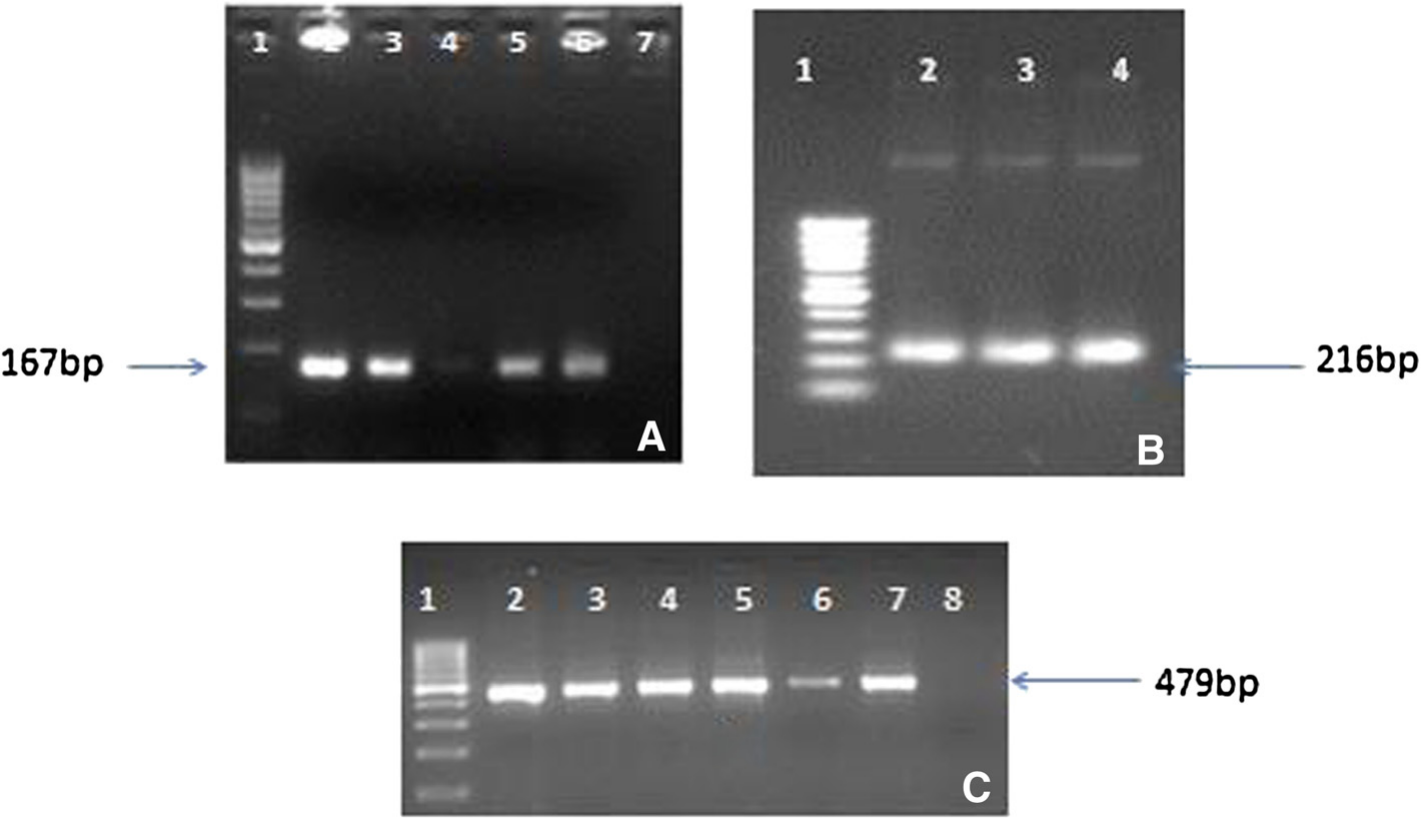
**Confirmation of successful cloning by PCR in Figure A Lane 1: 100 bp Leader Lane 1–6 PCR product of** ***Cry2A*** **Lane 7: Negative Control while in Figure B Lane 1: 100 bp Ladder and Lane 2–4: PCR product of 216 bp and in Figure C Lane 1: 100 bp Ladder, 2–7 Amplified product of Cy1Ac and lane 8 is negative control.**

**Figure 4:**
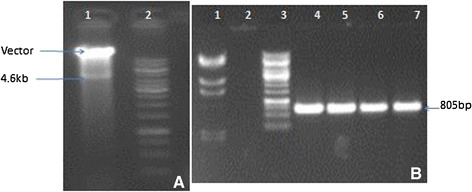
**A Lane 1 shows the digested product of 4.6 kb and Lane 2 shows 1 kb ladder whereas in B Lane one is lamda hindi-III ladder lane 2 is negative control, lane 3 is 1 kb ladder and lane 4–6 are PCR product of 805 bp.**

The transit peptide was isolated from Petunia (Figure 1). The construct MUX_01 was designed (Figure 2). Successful cloning was obtained by amplifying of 167 bp of *Cry2A*, 479 bp of *Cry1Ac*, and 216 bp of the transit peptide (Figure 3). The orientation was confirmed by specific primers, i.e., forward from *Cry1Ac* and reverse from *Cry2A*. An appropriate band of 805 bp and a digested product of 4.6 kb confirmed that *Cry1Ac* and *Cry2A* were successfully cloned in the correct orientation (Figure 4). The vector construction pattern is shown in a partial map (Figure 2).

### Transient expression through *GUS* estimation

**Figure 5:**
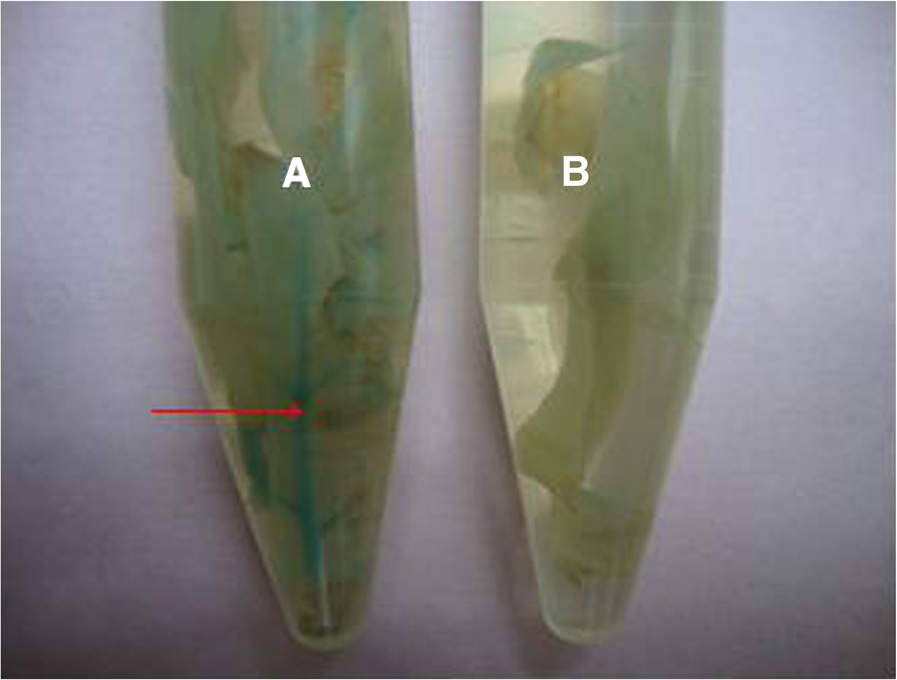
**Gus Expression in experimental plants. A**: transgenic plant leaves having blue-green color **B**: Non transgenic plant leaves as negative control with no color change.

**Figure 6:**
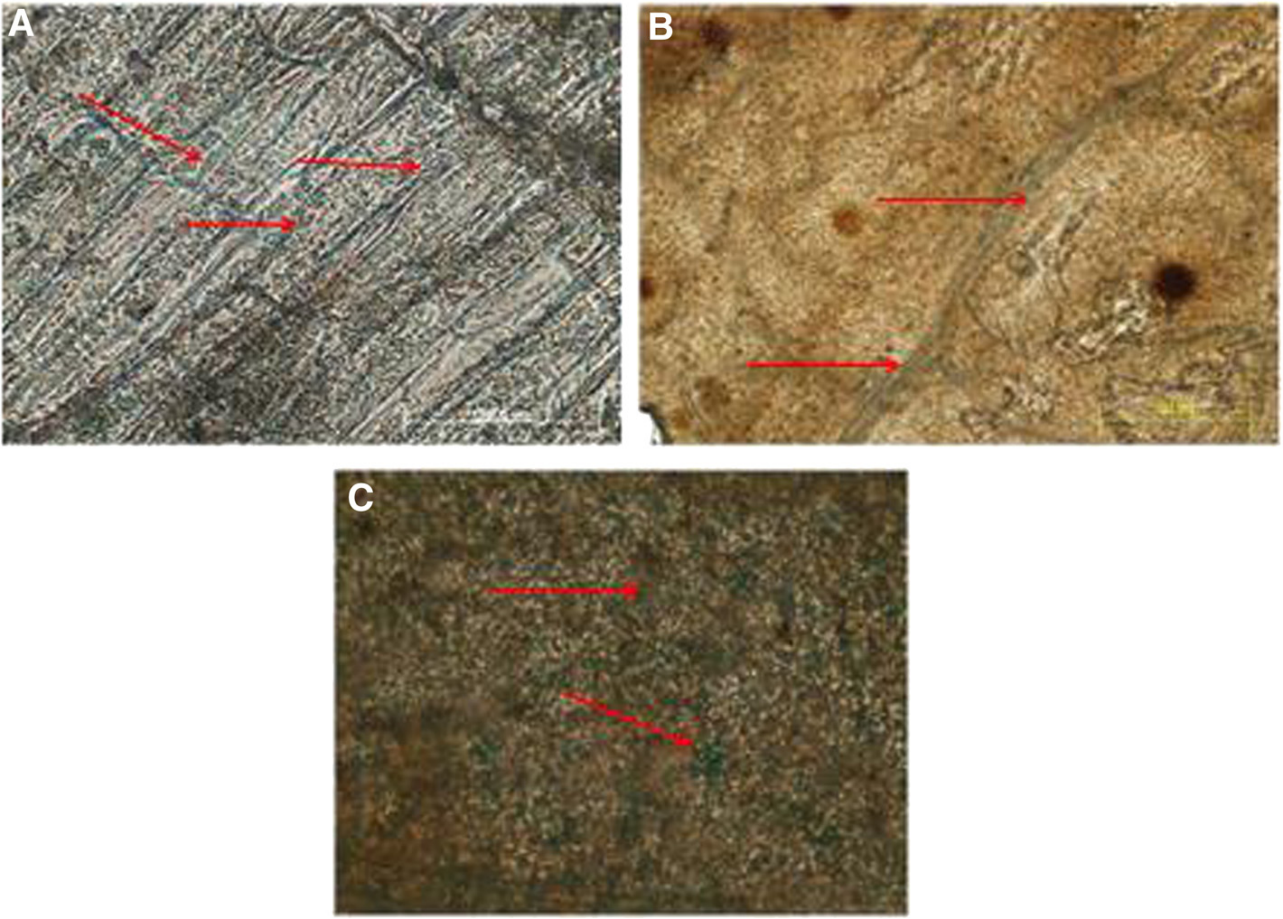
**Transient Gus expression. A**: In cotton stem section, **B**: Leaf midrib, **C**: In cotton leaf under florescent microscope. The bluish green color indicates the Gus expression.

A total of 1000 embryos were transformed with MUZ_01 (TP-*Cry1Ac* + CryIIA) and subjected to transient expression of the *GUS* gene. The appearance of a blue color in sections of infiltrated leaves after 72 hours confirmed the successful expression of the MUZ_01 construct vector in the plant expression system because *Cry1Ac*, *Cry2A*, and *GUS* gene expression were under the same promoter (Figures 5 and 6). A bluish green color was apparent in transgenic embryos but not in nontransgenic ones (Figure 6). Thirty plants that survived and passed screening were moved to selection free medium. In the end, seven plants survived soil acclimatization and were moved to the field. The overall transformation efficiency was calculated to be 0.7% (Table 1).

**Table 1:** **Transformation efficiency of Muz-01 Construct in Cotton**

**Construct**	**Germinated embryos used**	**Survival (3 weeks)**	**Survival (8 weeks)**	**Shifted in soil**	**Transformation efficiency (TE)%**
**pBI-121**	1000	129	36	7	0.7

### Molecular analysis of the putative transgenic plants

#### PCR analysis of putative transgenic plants

**Figure 7:**
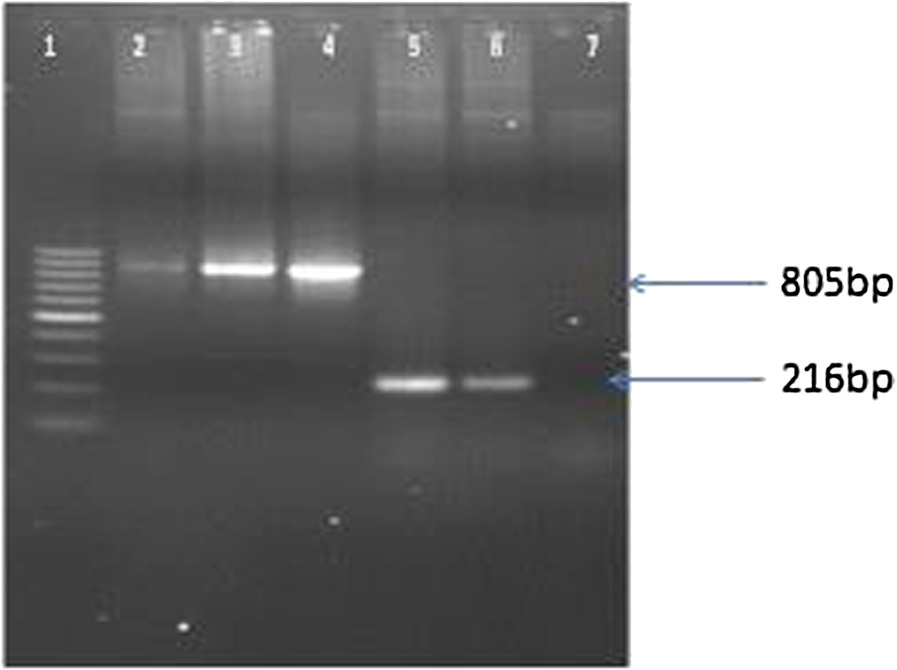
**Confirmation of Transgenic plants by PCR with orientation (** ***Cry1Ac*** **+** ***Cry2A*** **) primers and Tp2 primers.** Lane 1: 100 bp Ladder Lane, 2–3 PCR products with orientation primers Lane 4 positive control Lane 5–6 PCR product with Tp2 primers and Lane 7 Negative control.

The amplification of 805 bp from *Cry1Ac*-*Cry2A* and 216 bp from *Tp2* showed successful integration of the target genes into the cotton plant genomes Figure 7. A *Tp2*-*Cry1Ac*-*Cry2A* plasmid was used as a positive control, while DNA extracted from untransformed plants was used as a negative control.

### Qualitative analysis of the Bt protein in transgenic plants

**Figure 8:**
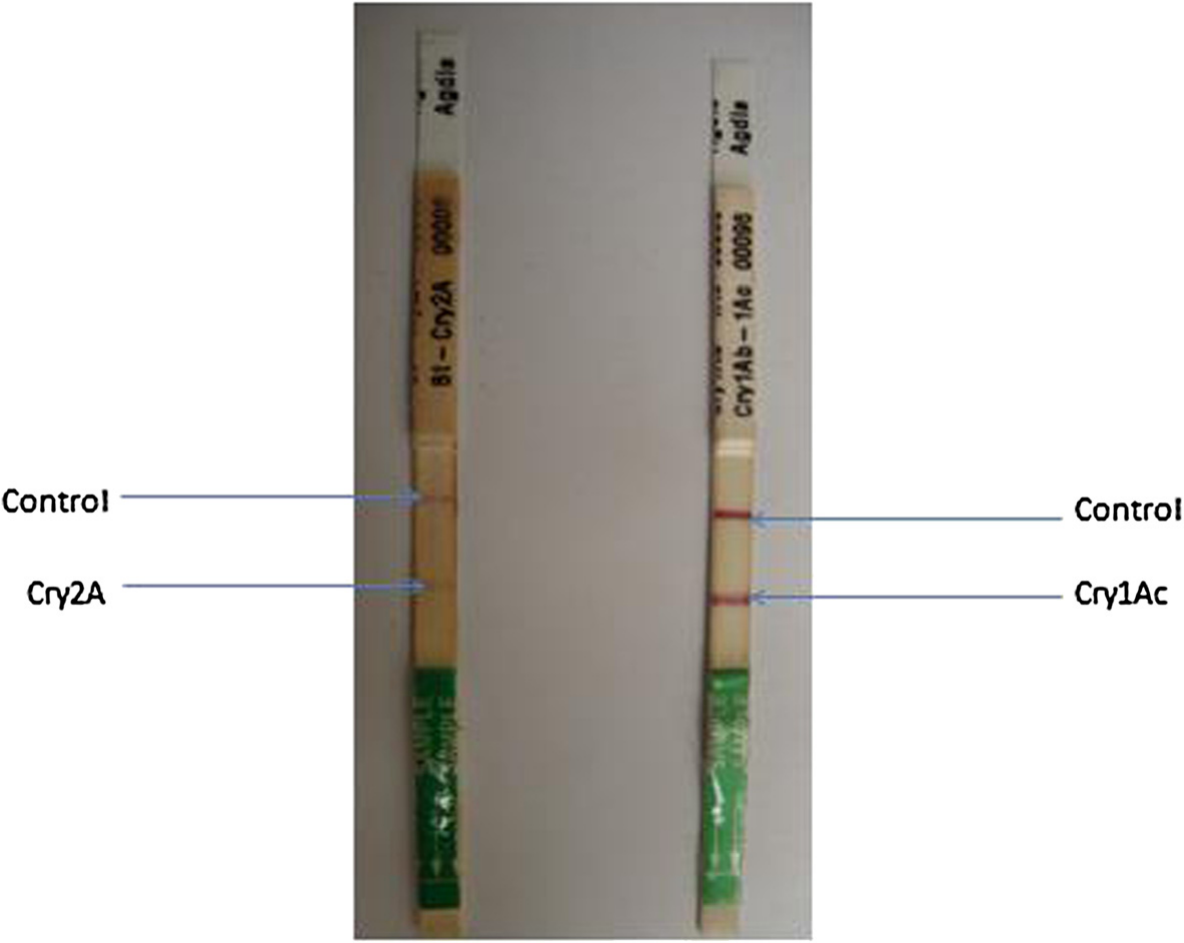
**Qualitative analysis of Bt protein in transgenic plants by using Dipstick assay.** Left row showing the concentration of Cry2A in μg/g while Cry1Ac in right row.

The polymerase chain reaction (PCR) confirmed transgenic plants were subjected to qualitative analysis through a dipstick assay. The presence or absence of the Bt protein in transgenic plants was confirmed by the presence or absence of bands at the test position along with a control band (Figure 8).

### Confirmation of MUZ_01 (Tp2- Cry1Ac-Cry2A) protein expression by ELISA

**Figure 9:**
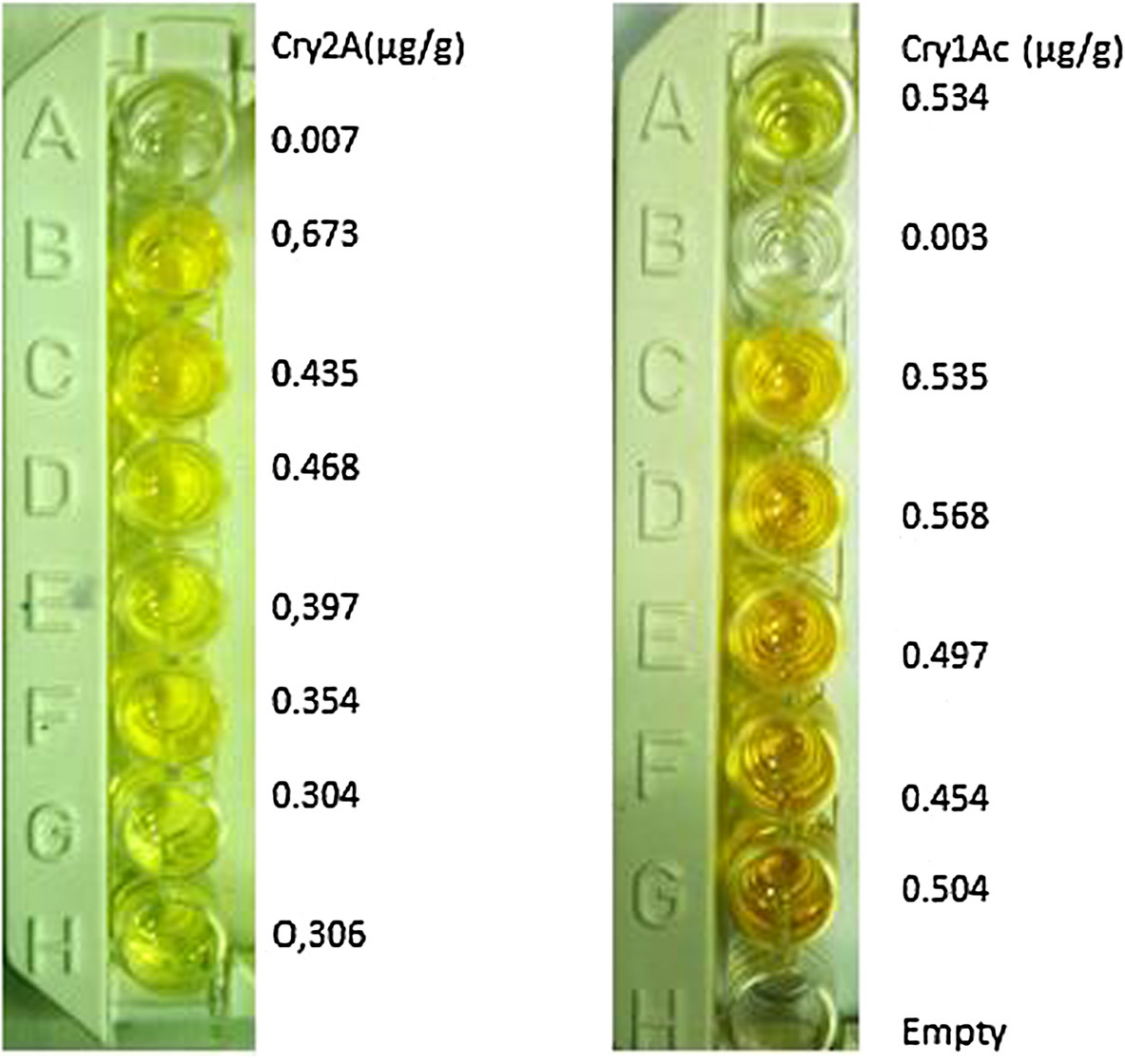
**Quantification of** ***Cry1Ac*** **and** ***Cry2A*** **protein by ELISA.**

Cry1Ac and Cry2A proteins in transgenic plants were quantified by enzyme-linked immunosorbent assay (ELISA) with an Envirologix Kit (Cat # AP051, 500 Riverside Industrial Parkway Portland, Maine 04103–1486 USA). Positive and negative controls were added to the wells along with test samples. ELISA was performed according to the manufacturer’s instructions and the endo-toxin (*Cry1Ac* and *Cry2A*) values were quantified as μg/g of fresh tissue [24] as shown in Figure 9. A maximum of 0.673 μg/g tissue of Cry1Ac and 0.568 μg/g tissue of Cry2A was observed in transgenic plants, while no Bt protein expression was observed in the nontransgenic control plants.

### Insect bioassay

**Figure 10:**
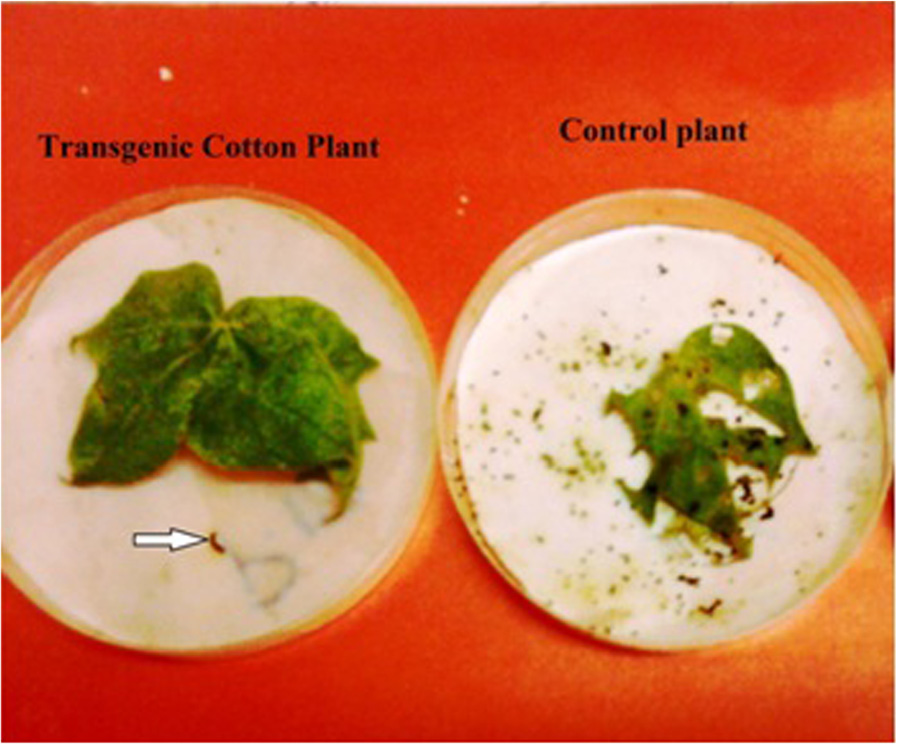
**Bioassy with American bollworm.**

Insect bioassays of transgenic and control plants were carried out in controlled conditions by simply using fresh leaves from transgenic cotton plants, along with nontransgenic control plants. We achieved 100% mortality in the target insect after 72 hours of feeding the 2^nd^ instar insect larvae with transgenic plants, while 100% survival on nontransgenic leaves determined the efficacy of the *MUZ_01* gene construct against the target insect pests (Figure 10).

### Phase contrast fluorescence microscopy

**Figure 11:**
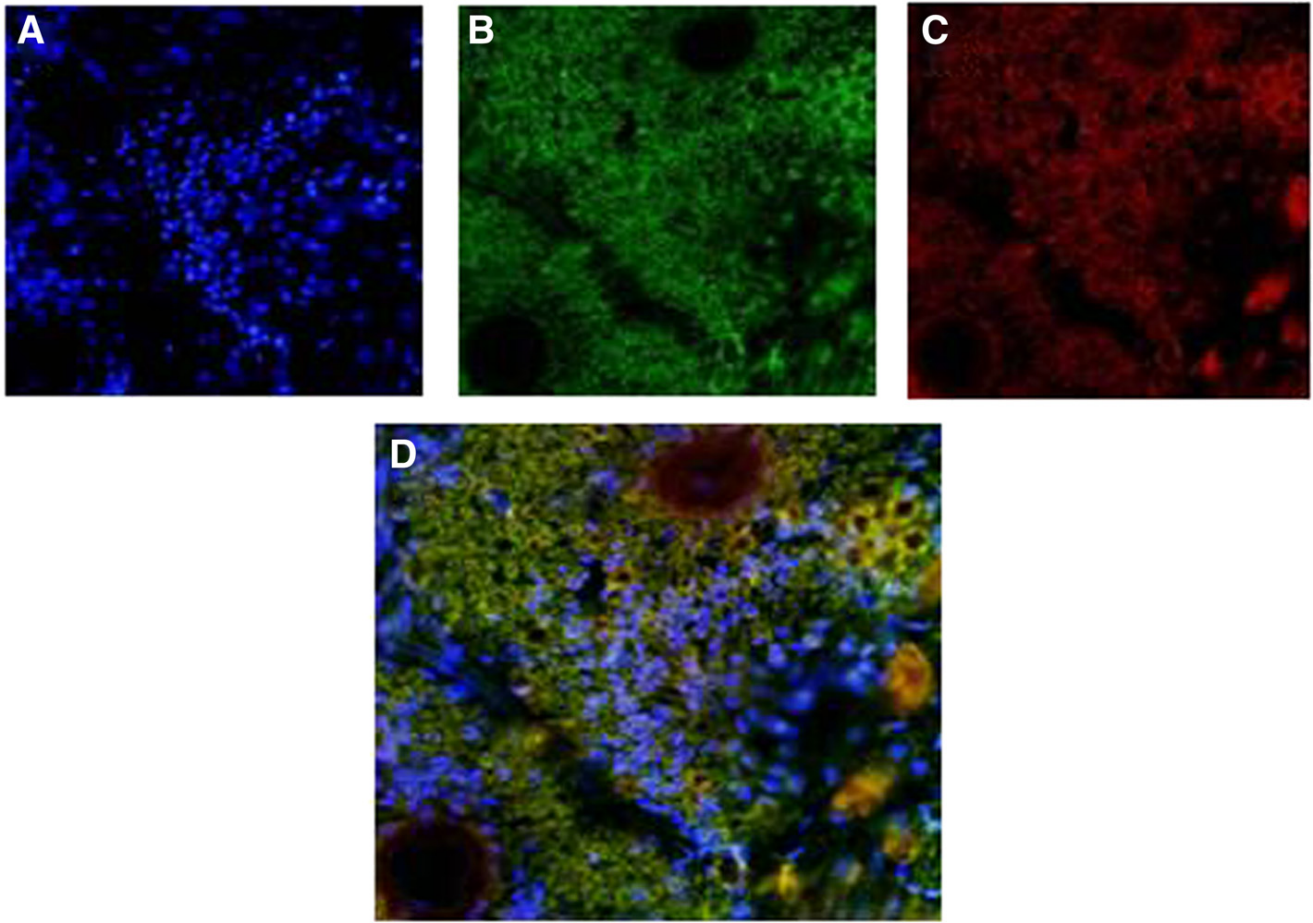
**Phase contrast fluorescence microscopy of** ***Cry1Ac*** **transgenic plants without transit peptide. A** = DAPI blue fluorescence. **B** = FITC green fluorescence. **C** = Chloroplast auto-fluorescence red. **D** = Merged image of I, II & III. Green red and blue colors do not merge i.e. *Cry1Ac* is outside the chloroplast.

**Figure 12:**
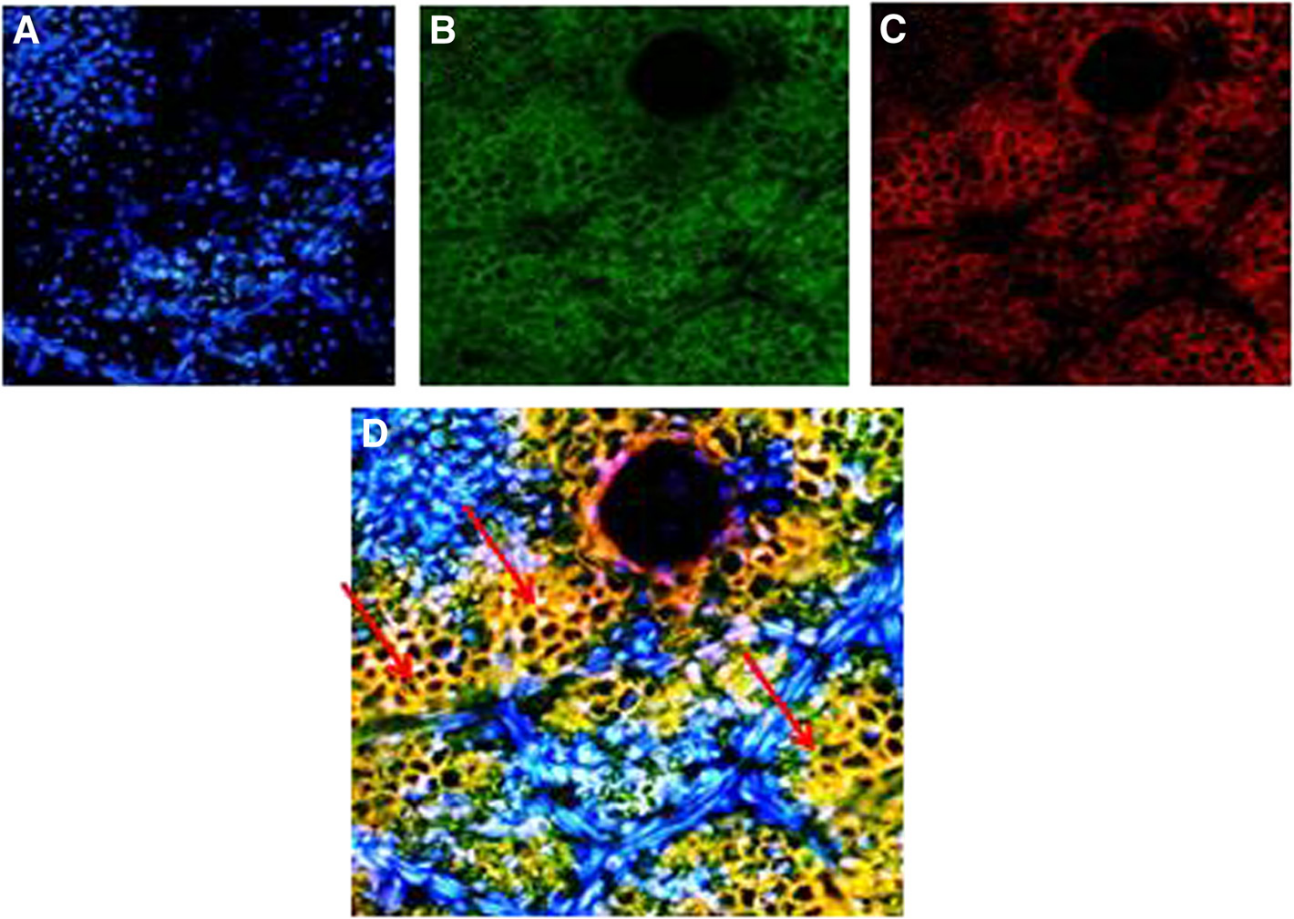
**Phase contrast fluorescence microscopy of** ***Cry1Ac*** **transgenic plants with transit peptide. A =** DAPI blue fluorescence. **B** = FITC green fluorescence. **C** = Chloroplast auto-fluorescence red. **D** = Merged image of A, B and C. Yellow color is produced where green and red fluorescence occurred at the same place i.e. *Cry1Ac* inside chloroplasts.

**Figure 13:**
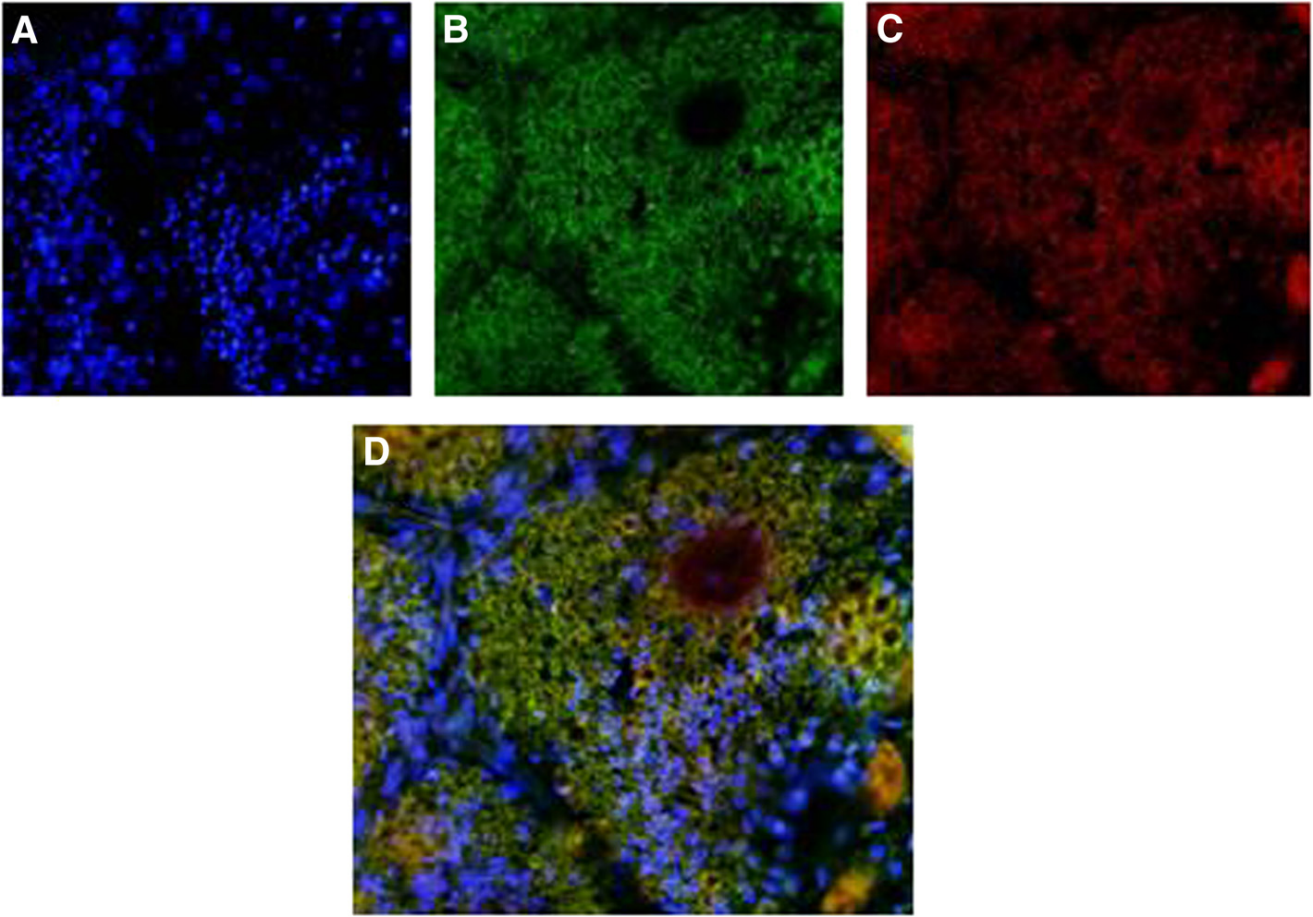
**Phase contrast fluorescence microscopy of** ***Cry2A*** **transgenic plants without transit peptide. A** = DAPI blue fluorescence. **B** = FITC green fluorescence. **C** = Chloroplast auto-fluorescence red. **D** = Merged image of I, II & III. Green red and blue colors do not merge i.e. *Cry2A* is outside the chloroplast.

**Figure 14:**
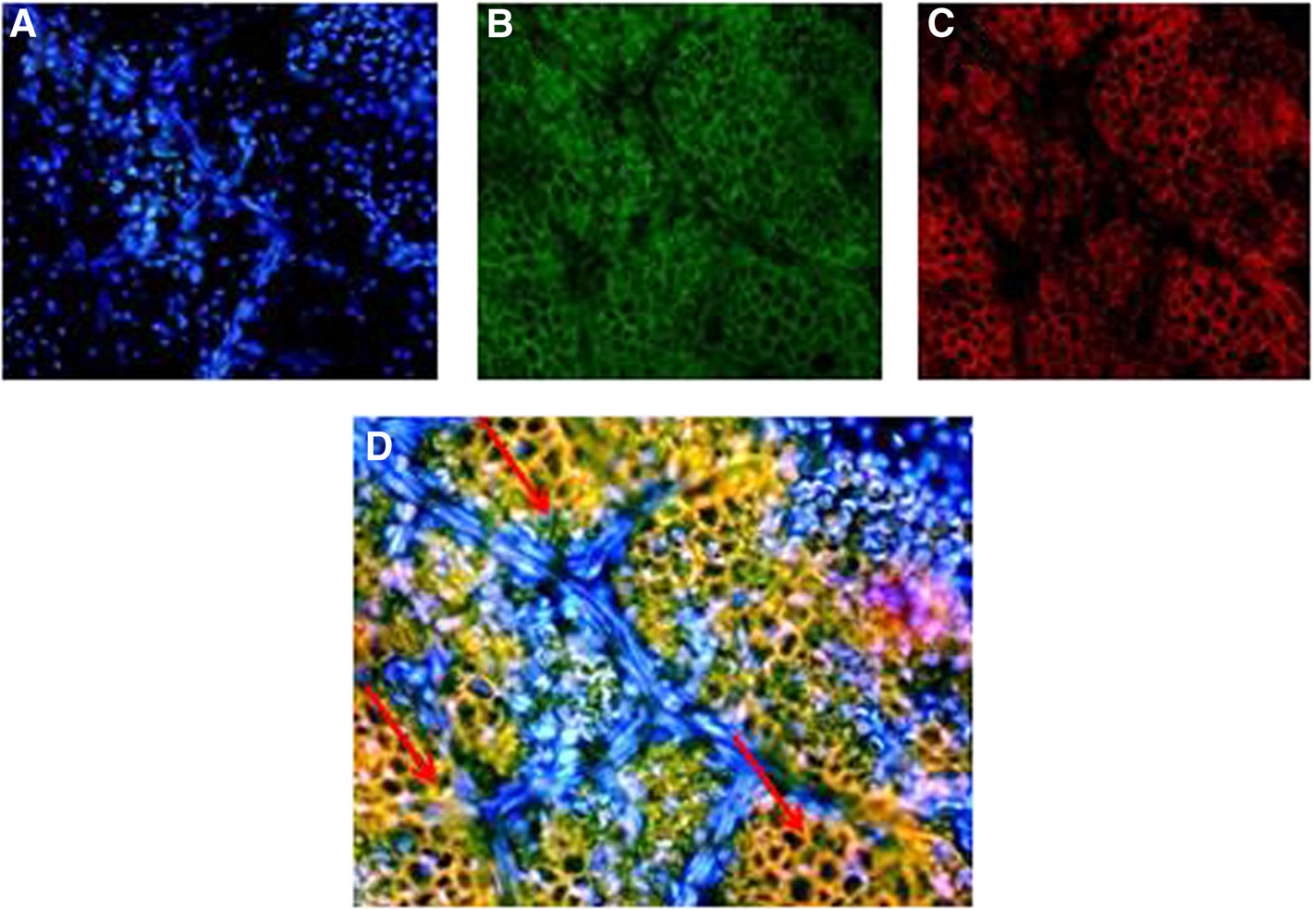
**Phase contrast fluorescence microscopy of** ***Cry2A*** **transgenic plants with transit peptide. A** = DAPI blue fluorescence. **B** = FITC green fluorescence. **C** = Chloroplast auto-fluorescence red. **D** = Merged image of A, B and C. Yellow color is produced where green and red fluorescence occurred at the same place i.e. *Cry2A* inside chloroplasts.

Fluorescein isothiocyanate (FITC) imaging of transgenic plants expressing *Cry1Ac*-*Cry2A* under cTP was taken at 488 nm and Chloroplast red auto-fluorescence at 580 nm excitation. Longitudinal leaf sections were labeled with primary antibody, anti-*Cry1Ac*/, anti-*Cry2A*, a secondary antibody, and FITC-conjugated IgG and observed under a phase contrast microscope (OLYMPUS DX61). 4’, 6-Diamidino-2-phenylindole (DAPI) was used to stain the nuclei. Cells stained with DAPI fluoresced blue, while those stained with FITC-conjugated IgG fluoresced green. The chloroplast itself gave off red auto-fluorescence, and the merged image of the transgenic leaves fluoresced yellow. In case of the control leaves, no yellow fluorescence was produced. These results indicate that Cry proteins were integrated into the chloroplast, i.e., transgenic plants under cTP, and in the case of the controls these proteins reside outside the chloroplast as indicated in Figures 11, 12, 13 and 14.

## Discussion

Chloroplast targeted expression of the Bt gene holds great potential for incorporating vital agronomic traits into plants. High Bt gene levels in chloroplasts permits plants to generate large quantities of crystal proteins. In the present study, two insecticidal genes, *Cry1Ac* and *Cry2A*, along with a chloroplast transit peptide were cloned in a PBI-121 vector and transformed into cotton variety MNH-786. *Cry1Ac* and *Cry2A* were selected because of their unique qualities, i.e., high expression levels and lack of competition for receptors among them.

The present study highlights the importance of cloning genes with transit peptides to demonstrate enhanced expression in cotton plants. *Cry1Ac* and *Cry2A* genes were cloned along with a chloroplast transient peptide in plant expression vector pBI-121 with the help of Hindi-III restriction sites. Successful cloning was confirmed by gene specific and orientation primers [25]. An agro-infiltration assay was used to check the efficacy of the cloned genes transient expression [11], resulting in a bluish-green color in the infiltrated region. Similar results were obtained by Ashraf, Bakhsh, and Pathi [26-28]. Transformation of the *Cry* genes with transit peptides not only makes it possible to localize transgene proteins in green parts of the plant, but it is also helpful in overcoming health and biosafety issues. Transgene protein expression was analyzed both qualitatively and quantitatively. Appearance of a band on the dipstick along with a control confirmed the presence of the target protein in the transgenic plant; similar results were reported by Dangat [29]. ELISA was performed to quantify the Cry proteins according to Li [30]. Maximum (0.673 ng, 0.454 ng) and minimum (0.306 ng, 0.568 ng) Cry1Ac and Cry2A proteins, respectively, were estimated in Muz-01 transgenic plants compared with control plants, which exhibited no Cry protein expression. Successful integration of Cry proteins into the chloroplast was confirmed by florescence microscopy [31] and FITC. Similar results for AtTrx-h3 expression in chloroplasts have been reported, while [32] used a similar technique for RB-60 protein expression in the cytoplasm and chloroplast. Insect bioassays revealed high mortality in the American boll worm. To check the efficacy of the transgene in the field an insect bioassay was performed. We recorded 100% mortality in the insects after feeding on the transgenic leaves, these results are comparable to those of Kiani [11].

From the above it is clear that the cloning of more than one gene, i.e., fusion genes, with transient peptides to localize the expression of these genes in the chloroplast not only increases the efficacy of the Bt proteins to kill the insects but it is also helpful in solving the biosafety concerns. On the basis of our molecular analysis we conclude that the transgenic plants with double Bt genes and a transit peptide for chloroplast expression was an excellent improvement in lepidopteron insect resistance. Our results suggest that transgenic cotton with transit peptide fusion protein genes is necessary to improve resistance against insects when compared with other genes without transit peptide fusion proteins.

## Conclusions

This investigation suggests that insect resistance in cotton by modifying cotton plant genetics with gene transformation is possible. We found that protection against insects was improved by integrating some of the unique features of chloroplast transit peptides into the cotton crop. The present study was designed to produce modern transgenic cotton plants with no biosafety concerns as the transgene is only expressed in green tissues because the fusion protein gene only attaches to Bt in the C-terminal and cTP in the N-terminal. Thus, the new transgenic cotton variety exhibits greater insect resistance and enhanced Bt expression only in the green parts of plants, which will result in reduced biosafety concerns and increased cotton yield.

## Methods

### Selection of plant materials

The transit peptide was first reported in petunia [Accession no. JF499829], which was locally available. For the isolation of cTP, petunia cultivar, *Grandiflora*, seeds were grown in the Center of Excellence in Molecular Biology CEMB green house at 25°C. Tobacco and cotton plants were also selected for transient expression and transformation, respectively.

### Isolation of the chloroplast transit peptide (cTP)

Total RNA was extracted from petunia leaves. Oligo (dT) 18 primers and the MMLuV-RT enzyme were used for cDNA library synthesis. An NcoI restriction site was used with the forward (TTAGCCATGGATGGCACAAATTAACAACATGG) and reverse primers (TAAGCCATGGCTGTGCTGTAGCCACTGATGC) to amplify a 216 bp fragment of the *TP* gene from the cDNA library. The amplified PCR product was cloned into a TA-vector PCR 2.1 (Invitrogen, Carlsbad, CA, United States of America). TP Sequencing was carried out with M13 primers on an ABI 310 Genetic Analyzer. Vector sequences were deleted in GeneDoc software.

### Plant expression vector construction

The CaMV35S-*Cry1Ac*-NOS cassette (2476 bp) was excised from a pK2Ac vector. This excised cassette was then purified and cloned into a pTZ57 vector to overcome the NcoI constrain. TP was then digested with NcoI and ligated towards the *Cry1Ac* N-terminal. A 35S-TP-*Cry1Ac*-NOS cassette was then cloned into pBI121 using HindIII restriction sites. *Cry2A* was digested with XhoI and treated with S1 nuclease to remove single-stranded overhangs and was then ligated towards the *Cry1Ac* C-terminal, which generated TP-*Cry1Ac*-Cr2A (Muz-01 name given to this vector). The correct *Cry2A* orientation was confirmed through PCR with orientation primers. The *Cry1Ac*-*Cry2A* orientation primers were: forward primer (CAGCAGTGGAAATAACATTCAGA) and reverse primer (AGCCTGTTGAGGAAGAGCTG), to give 805 bp amplification products.

### Confirmation of successful cloning

For the confirmation of successful cloning, the construct was checked by orientation PCR and restriction-digestion. A pair of primers was designed for the orientation PCR. The forward primer (CAGCAGTGGAAATAACATTCAGA) was designed from *Cry1Ac*, while reverse primer (AGCCTGTTGAGGAAGAGCTG) was designed from *Cry2A*. Following PCR amplification with orientation, successful cloning and the correct orientation were further confirmed by digestion and ligation. The Hindi-III enzyme was used for digestion and ligation. The Hindi-III enzyme digested the complete *TP2*-*Cry1Ac*-*Cry2A*-NOS cassette, thus releasing a 4.6 kb fragment. Therefore, the restriction-digestion and orientation PCR confirmed successful cloning and that the genes were cloned in the correct orientation.

### *GUS* leaf infiltration assay

*Muz-01* was transformed into *Agrobacterium tumefaciens* (LBA4404 strain) by electroporation. The efficacy of the construct vectors was confirmed by an Agrobacterium-mediated leaf infiltration *GUS* assay in both tobacco and cotton fresh leaves. The underside of the leaf was gently rubbed to remove the wax cuticle. *Agrobacterium* samples were taken with 5 mL syringes; the needle was removed, placed on the underside of the leaf, and pressed gently. Liquid diffused into mesophyllar air spaces. The infiltrated area was marked and tagged. These leaves were left for 72 hours under natural conditions and then subjected to a *GUS* assay.

### Detection of *GUS* activity

*GUS* activity in the infiltrated leaves was detected histochemically. The infiltrated portion of the leaves was excised and incubated in *GUS* staining solution (0.08% w/v X-Gluc in 0.1 M sodium dihydrogen phosphate pH 7.0, 0.2 mM 10% Triton, and 20% methanol) at 37°C. After staining with *GUS* solution these plant tissues were immersed in fixative solution, which consisted of formaldehyde (5%), ethanol (20%), and acetic acid (5%) for 10 minutes. To remove chlorophyll, the leaves were submerged in 70% ethanol for 48 hours. *GUS* activity was then observed by sight as well as under a florescent microscope (OLYMPUS SZX7).

### Cotton construct transformation

Cotton (*G. hirsutum*) cv. MNH 786 was selected for transformation because of its high yielding potential and susceptibility to lepidopteran insects. Delinted seeds were sterilized with Tween 20 for 4 minutes and then subjected to 0.1% HgCl2 and 0.1% sodium dodecyl sulfate. Sterilized seeds were placed in a seed germinator at 30°C overnight in the dark. Germinated seedlings were used for *Agrobacterium*-mediated transformation as used by [33] and modified by [24,34-36] at CEMB. MS medium [37] was used to culture the inoculated plants. Furthermore, 1 mg/l kinetin and 250 mg/l cefotaxime were used in the MS plates for the first 3 days, and after that the plantlets were subcultured in MS tubes containing 250 mg/l kanamycin, 0.5 mg/l benzylaminopurine, and 1 mg/l a-naphthaleneacetic acid. The putative transgenic plants were then moved to pots containing soil of equal proportions of clay, sand, and peat moss (1:1:1). Finally, the plants were moved to a greenhouse and subjected to various molecular analyses.

### Genomic DNA isolation and polymerase chain reaction

Genomic DNA was isolated from the putative transgenic cotton leaves according to the method of Zhang [38]. Successful integration of the genes into the cotton genome was confirmed by PCR amplification with internal *Cry1Ac* and *Cry2A* primers.

### Whole-leaf protein extraction

Whole-leaf protein was extracted from fresh transgenic cotton leaves. The leaves were crushed in liquid nitrogen. Ground leaves were put in 1.5 mL micro tubes with 400 μL of protein extraction buffer (1X). The samples were vortexed to homogenize and incubated at 4°C for 2 hours. The samples were then centrifuged for 10 min at 13,000 × *g*. The supernatant was eluted and stored in new 1.5 mL tubes and Bradford reagent was used to quantify proteins [39].

### ELISA

Leaf samples were ground in liquid nitrogen with a pestle and mortar. After grinding, 300 μl of protein extraction buffer was added and it was incubated at 4°C for overnight. The next day the mixture was centrifuged at 13,000 × *g* for 10 minutes. The supernatant was eluted. Fifty microliters of *Cry1Ac* and *Cry2A* enzyme conjugate was then added to each well immediately followed by 50 μl extraction buffer, 50 μl *Cry1Ac* and *Cry2A* positive control, and 50 μl of the sample extract to the their respective wells. The contents were thoroughly mixed by moving in a rapid circular motion for 20–30 seconds. The wells were then covered with parafilm and incubated at ambient temperature for 2 hours. After incubation the cover was removed and the wells were washed three times with washing buffer. Water was removed and 100 μl of substrate was added to each well. The wells were covered with parafilm and the plate was incubated at ambient temperature for 30 minutes. After incubation, 100 μl of the stop solution was added, turning the well contents yellow. Then wavelength of the spectrophotometer was adjusted to 450 nm and the readings were recorded.

### Insect bioassay

*Heliothis* larvae were employed for the insect bioassay [35,40] to examine whether or not the chloroplast targeted expression of the *Cry1Ac* and *Cry2A* fusion gene increased the *Heliothis* mortality. Larvae were collected from a CEMB field and used under laboratory conditions in a feeding bioassay. Leaves of both the control and transgenic cotton were placed in a petri dish, and larvae were allowed to feed on them. The leaves were examined after 48 hours.

### FITCH

For immunohistochemistry, the leaves were washed with 1X PBS twice and fixed with 4% paraformaldehyde solution. The samples were then incubated with primary antibodies, Anti-*Cry1Ac* and anti-*Cry2A* in a dilution of 1:100 for 1 hour at 37°C in a humidified chamber. Incubation with primary antibodies was followed by three washes with 1X PBS. The samples were then incubated with secondary antibodies specific to each of the respective primary antibodies and stained with DAPI (Invitrogen™, CA, USA) for 1 hour at 37°C in a humidified chamber. Images were taken for each group from three separate experiments using a phase contrast microscope (OLYMPUS DX61).
